# A model-based time-reversal of left ventricular motion improves cardiac motion analysis using tagged MRI data

**DOI:** 10.1186/1475-925X-7-15

**Published:** 2008-05-19

**Authors:** Tareq Alrefae, Irina V Smirnova, Larry T Cook, Mehmet Bilgen

**Affiliations:** 1 Physics, Kuwait University, Khaldia, Kuwait; 2 Physical Therapy and Rehabilitation Science, The University of Kansas Medical Center, Kansas City, Kansas, USA; 3 Radiology, The University of Kansas Medical Center, Kansas City, Kansas, USA; 4 Radiology and Radiological Science, Medical University of South Carolina, Charleston, South Carolina, USA

## Abstract

**Background:**

Myocardial motion is an important observable for the assessment of heart condition. Accurate estimates of ventricular (LV) wall motion are required for quantifying myocardial deformation and assessing local tissue function and viability. Harmonic Phase (HARP) analysis was developed for measuring regional LV motion using tagged magnetic resonance imaging (tMRI) data. With current computer-aided postprocessing tools including HARP analysis, large motions experienced by myocardial tissue are, however, often intractable to measure. This paper addresses this issue and provides a solution to make such measurements possible.

**Methods:**

To improve the estimation performance of large cardiac motions while analyzing tMRI data sets, we propose a two-step solution. The first step involves constructing a model to describe average systolic motion of the LV wall within a subject group. The second step involves time-reversal of the model applied as a spatial coordinate transformation to digitally relax the contracted LV wall in the experimental data of a single subject to the beginning of systole. Cardiac tMRI scans were performed on four healthy rats and used for developing the forward LV model. Algorithms were implemented for preprocessing the tMRI data, optimizing the model parameters and performing the HARP analysis. Slices from the midventricular level were then analyzed for all systolic phases.

**Results:**

The time-reversal operation derived from the LV model accounted for the bulk portion of the myocardial motion, which was the average motion experienced within the overall subject population. In analyzing the individual tMRI data sets, removing this average with the time-reversal operation left small magnitude residual motion unique to the case. This remaining residual portion of the motion was estimated robustly using the HARP analysis.

**Conclusion:**

Utilizing a combination of the forward LV model and its time reversal improves the performance of motion estimation in evaluating the cardiac function.

## Introduction

Cardiac dysfunction is associated with a variety of cardiovascular conditions that lead to heart failure. Significant cardiac events such as those in infarcted or diabetic hearts are associated with impaired relaxation and contraction of myocardium or abnormalities in left ventricular (LV) wall motion [1-5]. Tagged magnetic resonance imaging (tMRI) is an established cardiac imaging modality used to visualize regional myocardial motion within the LV wall [6] and references therein. Application of tMRI combined with sensitive motion estimation techniques, such as harmonic phase analysis (HARP), has proven to be feasible and diagnostically valuable in evaluating the performance of normal or diseased hearts in live subjects using conventional global and regional measures [7-11]. In the presence of large motions, however, the current analysis techniques including HARP fail to accurately describe the absolute displacement of the myocardial tissue. This paper addresses this issue and offers a solution – providing motion estimates with improved performance even when the tissue is subject to large movements. The solution requires characterizing the LV wall motion using a simple time-dependent model and performing time-reversal of tMRI data prior to analysis using HARP or other motion tracking techniques. In the following, we describe the forward LV model and give details of its implementation and algorithms involved during a priori data processing steps. Next, we explain the time-reversal operation. Using examples with real and simulated data, we demonstrate how the time-reversal approach improves the computer-aided regional myocardial motion measurements by minimizing the errors made during the digital estimation process.

## Background

### Tagged MRI

Tagged MRI employs electrocardiogram (ECG) gated acquisition. The imaging sequence contains initial saturation pulses followed by a repetitive image acquisition [6]. At a specific time point between the QRS peaks of the ECG waveform, spatial modulation of magnetization pulses is applied to saturate the spins perpendicular to the imaging plane in the body. Then, a series of images is acquired repetitively in equal time intervals covering the whole period of the heart beat. The resulting images provide snapshot views of the heart along either its short axis or long axis at different phases of the cardiac cycle. Depending on the nature and orientation of the saturation pulses, the first image in the series contains dark parallel lines, known as tags, which may be organized in horizontal, vertical or in both directions in a grid fashion. In the grid organization, the tag lines define boundaries of square-shaped cells. Thickness and separation of the tag lines are set to the desired values prior to the initiation of the data acquisition. As the heart contracts or relaxes, the tag lines follow the motion of the underlying myocardial tissue. The changes in relative distances between myocardial tissues as they move during the cardiac cycle define the tissue deformation. Measuring the regional motion and quantifying the resulting deformation experienced by each cell provides a sensitive measure to assess the viability of the myocardium within the cell.

### HARP analysis

Quantification of the actual deformation in the LV wall involves accurately measuring the motion and relative displacements experienced by the myocardial tissue. For measuring the cardiac motion, HARP makes use of the information embedded in Fourier spectrum of the tMRI [7]. The tag patterns give rise to spectral peaks, also known as harmonic peaks, in the spectrum. The peak corresponding to the fundamental frequency defined by the separation of the tagged lines is extracted by means of a band-pass filter. Two filters were designed to select the vertical or horizontal tag lines. Essentially, the inverse Hilbert transform of the filtered spectrum has two signal parts, namely magnitude *H *and phase *ϕ*. The resulting complex signal H can be written as H = *He*^*jϕ*^. While the magnitude is not of importance in motion quantification, the phase is considered to be the basis of the HARP analysis. The HARP is considered a material property of the moving cardiac tissue. Therefore, it can be used for tracking and quantifying the Lagrangian motion of myocardium.

HARP analysis requires two filtered complex image frames H_1 _and H_2 _from which the distribution of (either horizontal or vertical) displacement is estimated from the phase differences between using the formula

(1) $$\phi_{2}-\phi_{1}\;=∠\left({\text{H}}_{2}{\text{H}}_{\text{1}}^{\text{*}}\right)=∠\left(H_{1}H_{2}e^{j(\phi_{2}-\phi_{1})}\right)$$

where ∠ denotes phase angle and * denotes comlex conjugate.

## An outstanding problem in cardiac motion estimation

**Figure 1:**
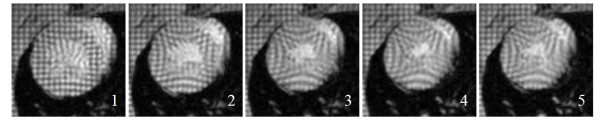
**Tagged MRI images of the LV in the short axis view are shown for the systolic part of the cardiac cycle (*i *= 1,..,5).** The myocardium exhibits relatively large displacements due to contraction.

**Figure 2:**
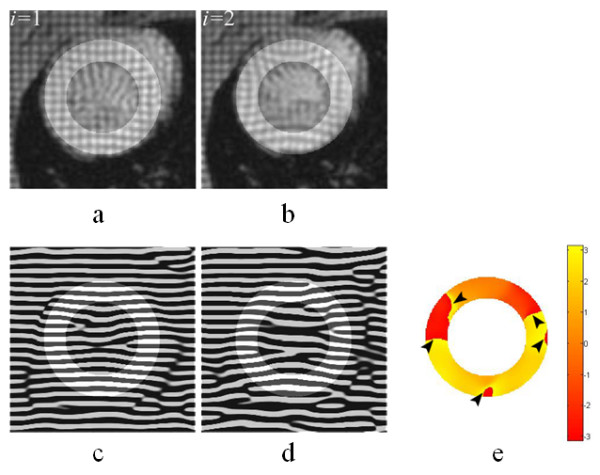
**Vertical motion of large magnitude between two image frames (a) *i *= 1 and (b) *i *= 2 with the corresponding vertical phase maps in (c) and (d) gives rise to phase wraps in (d) HARP-based motion map (black arrows point at phase-wrapped regions) computed using Eq. (1).** A mask is superimposed on the intensity images to indicate the ROI. The color bar denotes phase change in the interval [-*π*, *π*].

During cardiac cycle, the LV wall experiences relatively large motions as seen in Fig. 1, which shows image frames acquired sequentially within the systolic phase of a cardiac cycle from a rat heart. Preprocessing steps employed to obtain this real tMRI data from a rat heart were described by the algorithm given in Appendix A. The images *I*_*i*_, *i *= 1 ... 5, depict the LV motion sequentially from end-diastole *i *= 1 to end-systole *i *= 5 in equally spaced time intervals. Under the circumstance of large motions, displacement measurements using HARP become a challenging task to perform. The computed phase difference (*ϕ*_2_-*ϕ *_1_) in Eq. (1) wraps within the interval [-*π*, *π*], as shown in Fig. 2 for the vertical motion map computed from the image set *I*_1 _and *I*_2 _in Fig. 1. The phase wrapping makes it inadequate to accurately describe the absolute motion using the HARP analysis. Unwrapping the phase offers a remedy for this issue, but its complex and computationally time-consuming implementation is often a drawback [12].

In addition, statistical errors made during the estimation of large motions increase with the magnitude of the motion. Although, sophisticated signal processing approaches may improve the estimation performance by minimizing such errors, these methods also demand significantly increased computation time, as in the case of motion tracking algorithms employing window-based search techniques. In the presence of large motions, signal decorrelation between two image frames becomes a prominent factor in determining the errors. That is, a severe decorrelation between two windowed signals subsequently contributes to a motion estimation outcome with poor performance [13-15].

### A potential solution

It is possible to compensate for the degrading effects of signal decorrelation when the tissue motion is large. In our previous work in elastography imaging, we introduced global and adaptive regional signal stretching methods with the ultimate goal of producing better performance in displacement estimation [14]. Signal stretching procedures employed in our earlier works were, however, directionally linear and uniform in space. In ultrasonic elastography imaging, we demonstrated that the estimation performance is improved after globally decompressing the post-compression ultrasonic wave field composed of a backscattered signal received by each element of an array transducer by the same stretch value equivalent to the applied strain on the sample. In the adaptive stretching approach, the post-compression signals localized to a region within the wave field were expanded or compressed to increase the signal similarity with the pre-compression signals. Here, we adapt a similar strategy and introduce a time-reversal operation to improve the performance of tracking the LV wall motion by processing the tMRI data. The adaptation first requires modeling of the forward motion of the LV wall during systole, which is described in Appendix B. The model mimics the motion of the myocardial tissue by utilizing a spatiotemporal nonlinear mapping operator, **T **(Eq. B1). The operator involves a time-dependent parameter *α *accounting for the temporal changes in the LV size in the short-axis view at the mid-ventricle level. The function of the operator **T **can be thought of as radially contracting the image canvas *I*_1 _by an amount defined by *α*.

## Time-reversal operation

**Figure 3:**
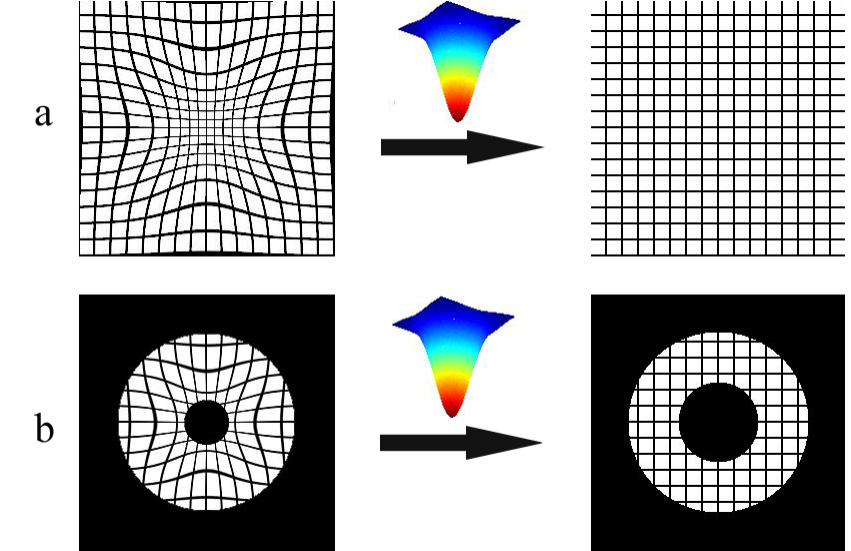
**Applying the operation **T^-1^**[**T**[*I*(*x, y*)]] on the deformed grid brings it back to an undeformed state (Fig. a). **Similarly, a deformed donut shaped disk is brought back to an undeformed state through application of time reversal (Fig. b).

Mathematically, the time reversal of the systolic forward motion of the LV can be obtained by the inverse operator **T**^-1^, i.e., the matrix-inverse of the forward motion operator **T**. Conceptually, the time-reversal operation can be considered as stretching the cardiac signals back in a radial fashion to the initiation of the systole. This effect is demonstrated in Fig. 3, where a deformed grid is restored to its pre-deformed state (Fig. 3-a), and a deformed tagged donut is brought back to its original undeformed shape (Fig. 3-b) by means of the time-reversal operation. Hence, in real MRI data, the forward model determined for the image frame *i *on the average can be operated in reverse, but on an individual image from a given set of sequential images. The result is a time-reversed pseudo image where the LV wall shape and size are expected to be closely similar to those seen in the image in frame *i *= 1, which is acquired at the beginning of the systole. In cause and event terminology, the time-reversal operation attempts to reverse a displacement event, by inverting its cause, namely the systolic contraction.

The true displacement **d **of myocardial tissue at a spatial location in the LV wall can be decomposed into two components:

(2) **d **= **Δ **+ **δ**.

Here, **Δ **denotes the bulk motion predicted by the time-reversal model. The term **δ **is the residual displacement that is not accounted for by the time-reversal operation and remains to be estimated between the image *I*_1 _and the time-reversed (TR) image. However, because the residual displacement is smaller in magnitude than the true displacement, its estimation results in small estimates with smaller errors when standard motion estimation algorithms are used.

To describe the above procedures in a compact form, we devise the following algorithm:

1. Obtain time-reversed image by applying **T**^-1 ^to the desired image frame *I*_*i *_with the corresponding *α*_*i*_.

2. Calculate the amount of bulk motion **Δ **analytically from **T**^-1^.

3. Estimate the amount of residual motion **δ **between the time-reversed image TR = **T**^-1 ^[*I*_*i*_] and the first systolic frame *I*_1 _using a method of preference for the motion estimation, e.g., HARP.

4. Add **δ **to **Δ **to get an estimate for the true total displacement **d**.

## Methods and procedures

### tMRI acquisition

Cardiac MRI data from the mid-ventricle level were collected from four male Sprague-Dawley rats. The rats were anesthetized using 1.5% isofluorane in a mixture of air and oxygen (60% and 40% respectively) and scanned using a 9.4 T horizontal bore scanner (Varian Inc., Palo Alto, CA) and 60 mm radio frequency volume coil. ECG gated gradient echo based tagged images were captured from the short-axis view of the heart. The cardiac cycle was temporally resolved into ten equally incremented phases. The first five were the systolic frames. The following settings were used for the image acquisition: repetition time = 25 ms, echo time = 2.44 ms, number of averages = 1, field of view = 60 × 60 mm, image matrix = 256 × 256, slice thickness = 2.0 mm. The square grid tags had dimensions of width = 0.3 mm and separation = 0.8 mm. All experimental procedures were approved by the University of Kansas Medical Center Institutional Animal Care and Use Committee.

### Construction of the LV motion model

**Figure 4:**
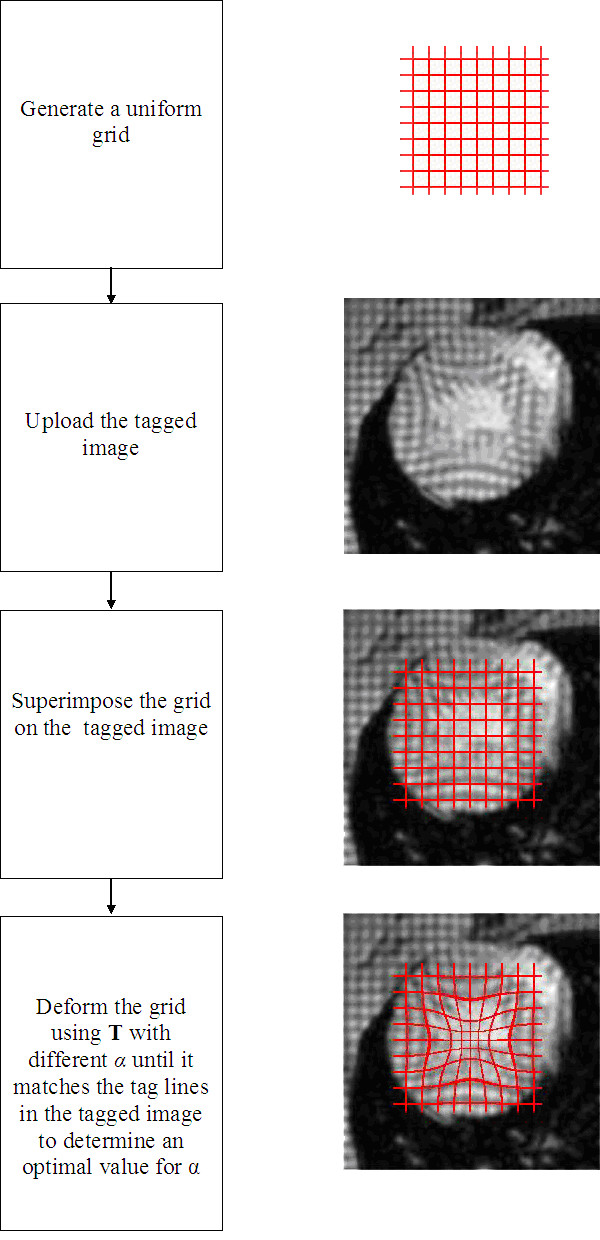
An algorithm to estimate an optimal value for the parameter *α *in the Gaussian transformation **T **using an image acquired at a systolic phase.

The characterization of the motion operator **T **from the real tMRI data requires finding *α*_*i *_values such that the computationally-deformed end-diastole image *I*_1 _of the LV matches the image *I*_*i *_in the sequence. The experimental data from each animal were preprocessed separately by following the algorithm described in Appendix A using all systolic frames. The resulting images were further processed to empirically find an optimal value for the deformation parameter *α*_*i *_for each systolic image frame *I*_*i *_for *i *= 2 ... 5. This process involved superimposing a computer generated grid of binary grid mesh, on the LV image as described by the algorithm in Fig. 4. The forward transformation **T **was applied to the undeformed mesh structure with a preselected *α*_*i*0 _value to deform the grid lines to match the tag lines in the image *I*_*i *_as close as possible. The preselection was made based on the numerical simulations performed earlier. Next, an iterative optimization procedure was implemented to achieve a best match by varying the parameter *α*_*i*_. This procedure involved converting the LV image *I*_*i *_to a binary form by applying an intensity threshold. The tag lines on the resulting image assumed zero intensity. Next, a logical XOR operation was performed between the binary image and the deformed grid mesh. The outcome of this operation in each position was 1 if the two pixel values were different, and 0 if they were the same. The results at pixels where the grid lines were collocated were summed. This sum was then normalized with the total number of pixels occupied by the lines on the grid mesh. A perfect match between the grid lines and the real tag lines would ideally produce a zero sum. This computational procedure was repeated for ten *α*_*i *_values distributed between a range divided in equal intervals; 0.9*α_*i*0 _<*α*_*ij *_< 1.1*α_*i*0_, j = 1 ... 10. The value of *α*_*ij *_producing the minimal normalized sum was selected as the optimal value for the deformation parameter *α*_*i *_for the frame *i*. When the normalized sum attained the same value for more than one *α*_*ij*_, one of these values was selected by visually inspecting the matches on the computer screen.

### Time-reversal operation

The time-reversal operation **T**^-1 ^was applied on an image frame *I*_*i *_with the corresponding optimal *α*_*i *_value. The resulting image TR was compared with the end-diastole image *I*_1 _and the HARP analysis was performed in between.

## Results and discussion

### Forward LV motion model

This study was initiated to seek a simple, yet comprehensive model with a sufficient number of elements to accurately represent the LV wall motion at the mid-ventricle level. With a set of basic assumptions and simplifying approximations, we proposed the transformation in Eq. (B1) to model the myocardial motion during the systole. We specifically targeted the mid-ventricle for modeling the LV motion because this level experiences minimal amount of torsion and twisting during the heart beat, allowing the model to only account for the radial motion of the wall. We empirically constructed the model parameters using real tMRI data gathered from the hearts of four rats. This implementation involved data processing steps, which were described algorithmically in Appendix A. The initial step involved windowing and scaling the images to consistently depict the LV size and shape with the same dimensions at the end-diastole. This approach made the model versatile enough to analyze the LV motion in different species or minimize the inter-variation of the measurements when the same species is considered. This feature of the model increases its capacity by simulating the LV motion in both humans and animals.

**Figure 5:**
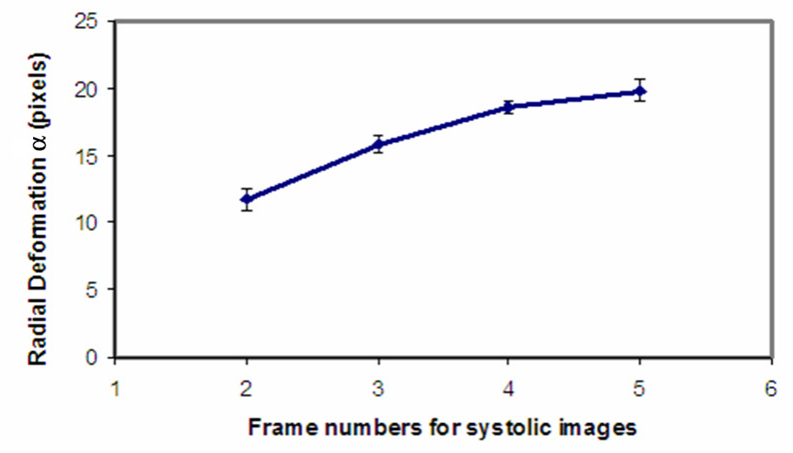
The deformation parameter *α *is plotted against the systolic image numbers, which are directly proportional to the acquisition time in the systolic phase. The curves in the graph exhibits a nonlinear behavior.

**Figure 6:**
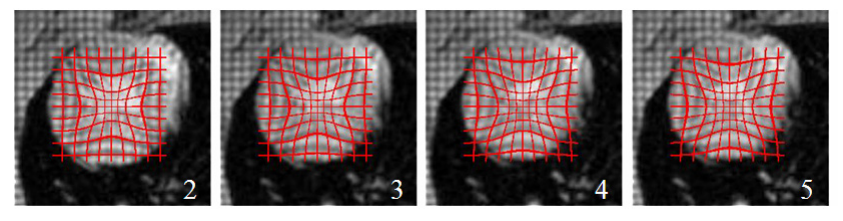
**Real time systolic images (2–5) of the left ventricle from Fig. 1.** Superimposed is a grid (red) that was deformed offline using the Gaussian transformation **T **applied on the image 1 according to the algorithm in Fig. 4. The grid (red) mimics the deformed tag lines in the underlying myocardial tissue. The estimated optimal *α *values are respectively 12, 16, 19 and 20.

Irrespective of whether the data is clinical or experimental, our LV model requires estimating a single deformation parameter *α *for quantifying the temporal motion in the systolic phase of the cardiac cycle with sufficient accuracy. Requiring only one parameter to reasonably describe the myocardial deformation is another important feature of this model. Table 1 presents estimates of *α *from the tMRI data obtained during the studies. The data in the table are also plotted against the frame numbers in Fig. 5. In light of the data presented in the figure, *α *increases with time in a nonlinear fashion to accommodate the shrinkage of the LV size during the systolic contraction. Figure 6 illustrates deformed grids (in red) superimposed on all four tagged images acquired serially during the systole. These results, i.e., the *α *values at different time points, establishes a normative database which may be used as reference for the comparison of results from tMRI experiments preformed on rats with abnormal cardiac conditions.

**Table 1:** The values (mean ± std) for the parameter *α *estimated using the data acquired from the control rats (*n *= 4).

Frame	*α *(pixels)
2	12.0 ± 0.8
3	16.0 ± 0.6
4	19.0 ± 0.4
5	20.0 ± 0.8

Because the MR sequence applies tagging gradients in the image frame *i *= 1(end diastole), the first noticeable deformation in tag lines occurs in frame *i *= 2.

Our model is also open to other clinical relevance. For example, strain calculation and its relation to the parameter *α *is a good area for future exploration in cardiac imaging research. Such a relation could, in principle, indicate a certain systolic dysfunction associated with certain diseases or abnormalities, such as diabetes. Similarly, another useful parameter well accepted by clinicians is the myocardial strain rate. By calculating the differences in strains exhibited by the LV tissues from one systolic phase to another, more insights may be gained regarding the myocardial behavior and its time dependence. These and other potential merits of the proposed motion model of the LV are left for future work.

**Figure 7:**
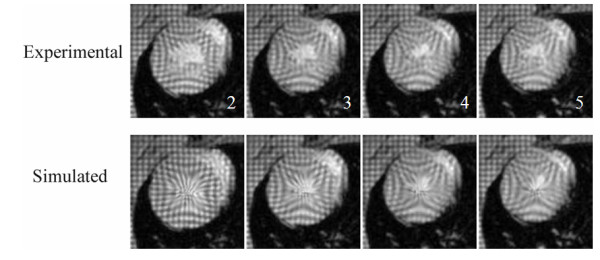
**Real and simulated pseudo images in systole.** The simulated images were obtained offline using the operation **T **on the real tagged image 1 with the optimal *α *values 12, 16, 19 and 20, as in Fig. 6. The goodness of fit between the real and simulated images were quantified using correlation coefficient. The coefficients were computed after masking out the LV wall on the images. The results were respectively 0.90, 0.94, 0.97 and 0.98, indicating high level of match between the intensity patterns within the LV wall between the pairs.

From another aspect, knowledge of the *α *values in Table 1 plays a role in applications where projecting the LV motion forward in time is required. By interpolating *α *in time, one can reconstruct simulated pseudo images corresponding to a specific time point chosen within the systolic phase of the cardiac cycle. These computational processes can be carried out offline by applying the transformation **T **to the canvas of the end-diastole image frame *i *= 1 with the desired *α *value. Sample results from such computations are shown in Fig. 7, where the simulated pseudo images are presented with their experimental real time counterparts for animal number 1. To evaluate the goodness of fit, we computed cross correlation coefficients between the real data and simulated systolic image pairs after segmenting out the LV wall with an identical mask. The coefficients were 0.90, 0.94, 0.97 and 0.98 for the frames 2, 3, 4 and 5 in Fig. 7, respectively. The measurements from other animals were given in Table 2. Combining the results together show strong correlation and confirm that the intensity patterns within the LV wall matched well between the real and simulated data. High level of goodness fits also imply that the torsion at the mid-ventricle level of heart is minimal, supporting our original assumption.

**Table 2:** The correlation coefficients computed to quantitatively assess goodness of fit between the real and simulated pseudo images for each rat.

Frame Number	Animal 1	Animal 2	Animal 3	Animal 4	Mean ± Std
2	0.90	0.82	0.63	0.96	0.83 ± 0.14
3	0.94	0.83	0.60	0.96	0.83 ± 0.17
4	0.97	0.83	0.61	0.97	0.85 ± 0.17
5	0.98	0.82	0.60	0.94	0.84 ± 0.17

For each frame, the simulated image was obtained by deforming the end diastole image with the forward model using the corresponding mean *α *value from Table 1. Note that the same masking function was used to segment the LV wall in real or simulated images, but the masks were different for each frame or animal.

A literature search reveals numerous approaches that have been employed to model the LV wall motion [16,17]. Examples include techniques based on finite elements, finite differences, B-spline methods, and prolate spheroidal basis functions [18-20]. Such techniques have attained good results in describing the cardiac wall motion and computing useful clinical data like displacement and strain profiles. Nevertheless, to accurately describe the LV wall motion, these models require large numbers of parameters and intense numerical computations. The need for a simple, yet reliable model has therefore been met by the current implementation.

### Limitations of the forward LV motion model

Despite its ability to describe the myocardial forward motion, our model suffers from two main limitations. The first limitation is that the model treats the entire myocardium as if it were contracting homogeneously. While such treatment greatly simplifies the problem, it ignores the heterogeneous nature of myocardial contractility. Evidently, contractility varies with the position of the myocardium such that tissues in the wall exhibit deformation profiles that differ from those of the septum. This variation in deformation is due to the fact that, depending on the location, some tissues have constraints that limit their motion while others do not. Accommodating this behavior with regional dependencies requires more complicated models to better describe the myocardial motion.

Another limitation of the model is its inability to handle twisting (torsion) motion that takes place in the basal and apical levels of the LV wall. Thus, the model is limited to work at the mid-ventricle level where the twist is minimal. Nonetheless, adding a twisting ability is plausible and computationally possible by having position-dependent angles to account for rotation in the transform operator in Eq. (B1). Such integrations are likely to increase the capability of the model to represent the myocardial motion at various levels of the LV in short axis views.

Although a typical cardiac cycle is composed of both systolic and diastolic phases, we chose to focus the model on systolic motion only. This approach was chosen to simplify the problem, knowing that we could easily extrapolate the analysis to include the diastolic motion if desired.

### Application of the time-reversal of the LV model to aid the analysis of the cardiac motion using HARP

**Figure 8:**
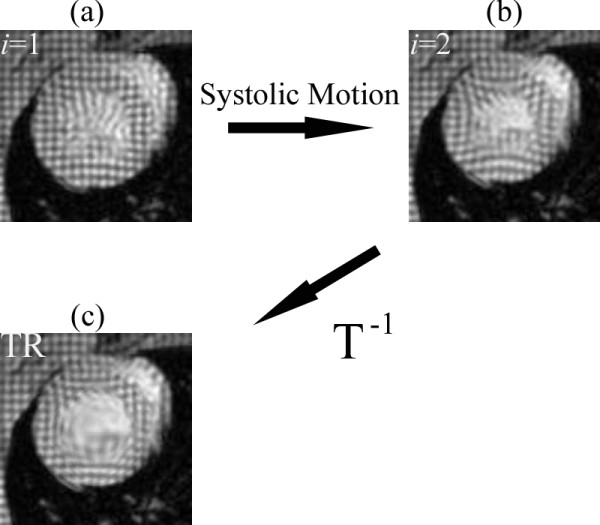
**Cardiac systolic motion changes image *I*_1 _(a) to *I*_2 _(b) causing large wall motion.** Applying time-reversal to *I*_2 _yields a time reversed image (c) with LV wall tissues almost restored to its initial positions in *I*_1_.

**Figure 9:**
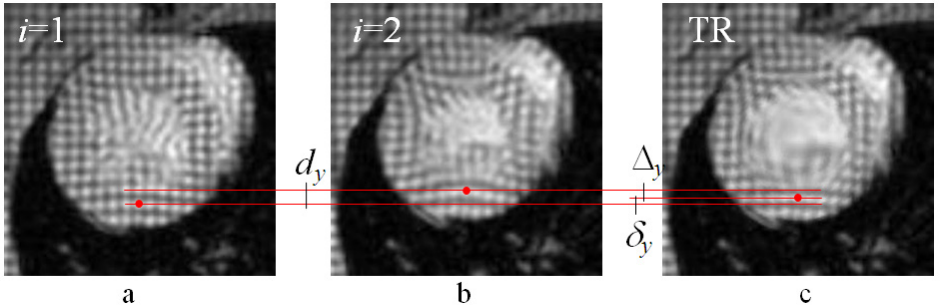
**Utilizing time-reversal, the bulk motion **Δ **between *I*_2 _and the time-reversed image TR is computed using simple matrix multiplication. **The residual motion δ between TR and *I*_1 _is estimated by a method of preference, such as HARP. The figure depicts the y components only.

Figure 2 shows the vertical motion map produced between the two image frames *I*_1 _and *I*_2 _using HARP analysis. The large motion between the frames is clearly seen to produce phase wraps. Figure 8 illustrates the same image frames *I*_1 _(Fig. 8-a) and *I*_2 _(Fig. 8-b) as well as the time-reversed image TR = **T**^-1 ^[*I*_2_(x, y)] of the frame *i *= 2 (Fig. 8-c). Comparing the intensity features indicates that the LV wall size and dimensions on the time-reversed image TR are nearly restored to those seen on the image *I*_1_, and the tag lines are straightened. Figure 9 depicts the images in Fig. 8 side-by-side and illustrates how a material coordinate in the LV wall moves with the real forward and time-reversed motions. The figure also depicts the vertical components of the displacement described by Eq. 2. The displacement of the tissue at the selected coordinates is seen much smaller in the (*I*_1_, TR) pair than it is in the (*I*_1_, *I*_2_) pair, making the former pair favorable for the motion calculations once the noise is taken into consideration.

**Figure 10:**
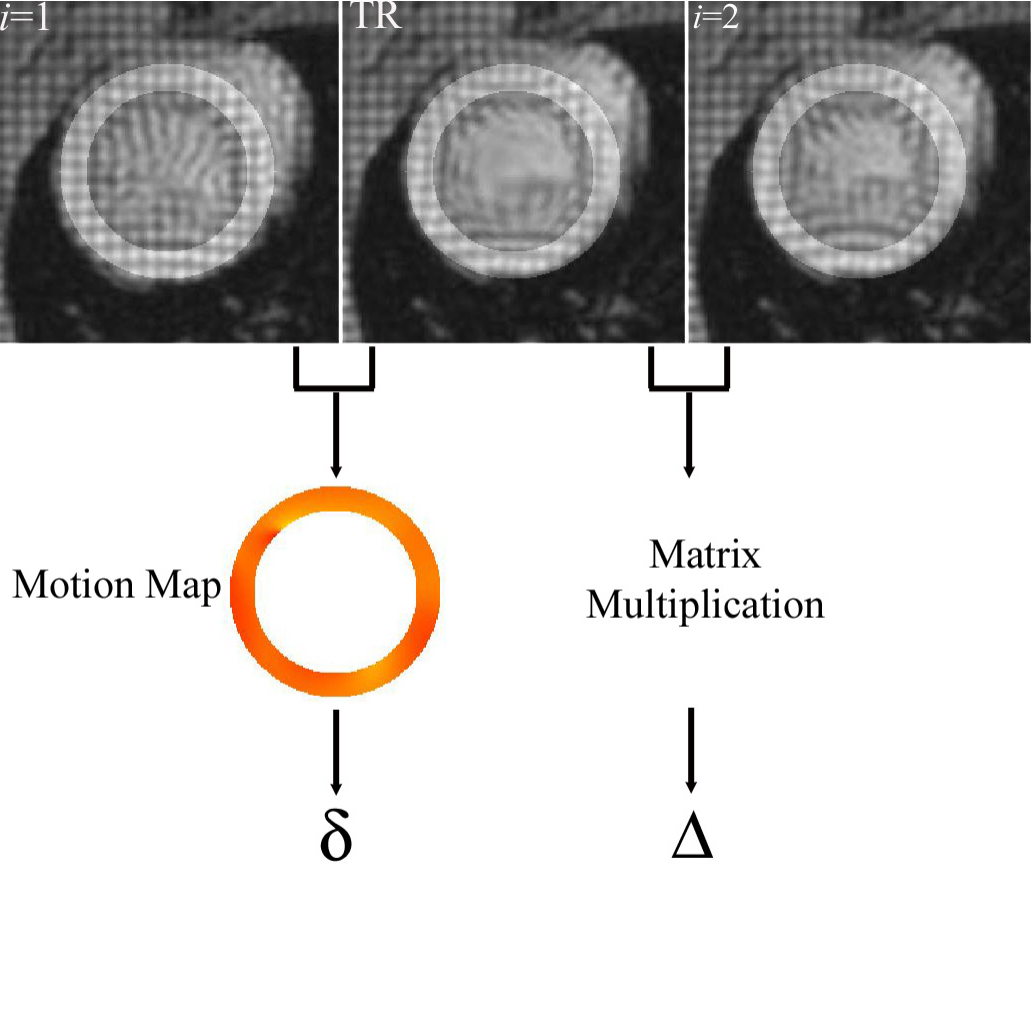
**Utilizing time-reversal to compute motion between the time-revered image TR and I_2_. **Thus magnitude of motion is considerably reduced between TR and I_1 _thereby yielding a phase-wrap free HARP motion map. A mask is superimposed on the image frames to indicate the ROI.

Figure 10 demonstrates the result of residual vertical motion $\tilde{\delta }$ computed between *I*_1 _and TR using HARP analysis. Here ~denotes estimate. This data shows that phase wrapping is no longer an issue in the vertical motion map. Reversing an event (displacement) by inverting its cause (systolic contraction) is seen as playing a beneficial role in improving the performance of the motion estimation with HARP. The bulk motion component **Δ **in Eq. 2 represents the average behavior of the subject population, and analytically available from **T**^-1^, for a given *α *value in Table 1. From the phase map in Fig. 10, **Δ **is primarily responsible for the resulting phase wraps, observed in Fig. 2, and its removal with the time-reversal operation is seen to leave the smaller magnitude $\tilde{\delta }$ component behind to be reliably estimated using the HARP analysis. We note that $\tilde{\delta }$ is unique to each subject, representing the sample-dependent inter-variation. **Δ **and $\tilde{\delta }$ are combined to reach the estimate of the final large motion $\tilde{d}$ between the image pair (*I*_1_,*I*_2_).

**Figure 11:**
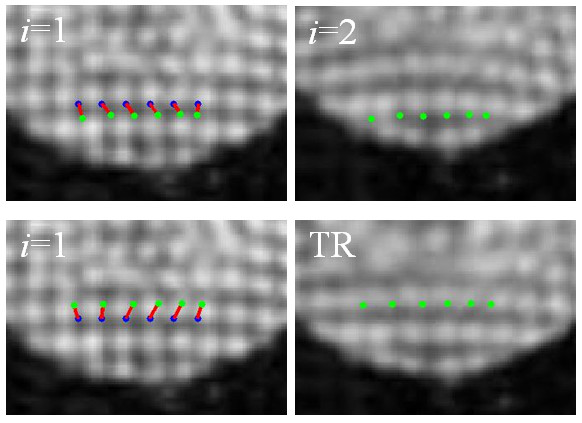
**Motion estimates for six selected points using HARP analysis between cardiac image frames 1 and 2 or frames 1 and TR. **Displacement vectors (red lines) start from the selected points (blue dots) and project to the end points (green dots). Please see the text for the detailed explanation of the figure.

Figure 11 shows an example and provides quantitative data, further demonstrating the role of the time reversal operation in improving the performance of motion estimator in HARP analysis. The figure shows cardiac image frames 1 and 2 on the top row and frames 1 and TR at the bottom row. Blue dots are the selected points on frame 1 and our goal is to estimate their displacements as they move with systolic contraction. Red lines denote the estimated displacements. Green dots denote the end points of the vectors. From the results, we see that all of the vectors computed using HARP analysis applied to frames 1 and 2 unrealistically behave as if the LV relaxing instead of contraction, and fail to follow the true displacement of the underlying cardiac tissue. This mismatch was mainly due to the limitation in the motion tracking capability of HARP analysis when the true motion between the sequential frames was large, causing the phase difference in Eq. (1) likely to wrap in the presented case. The time reversal operation did not exactly reverse the systolic LV wall motion, as were seen by the differences in the LV sizes on frames 1 and TR, but allowed the HARP analysis to produce reasonable data. When frames 1 and TR were used for the analysis, the vectors estimated correctly described the true motion. For the selected points from left to right as they appear on the image, the horizontal and vertical components of the vectors estimated from frames 1 and 2 were $\tilde{d}$ = (1.66,-6.16), (3.58,-4.6), (3.57,-5.14), (3.05,-4.75), (2.69,-4.34) and (-0.41,-4.71) pixels. The corresponding components of the vectors estimated from frames 1 and TR were $\tilde{\delta }$ = (-1.75,5.64), (0.38,5.74), (2.92,5.90), (3.45,6.29), (3.19,6.32) and (1.59,5.95) pixels. The components computed by the time-reversal model were **Δ **= (-3.23,-7.23), (-2.3,-7.95), (-1.08,-8.38), (0.27,-8.48), (1.59,-8.24) and (2.73,-7.69). This example and quantitative measurements clearly demonstrate the advantage of our approach. Preprocessing systolic frames with the time reversal operation prior to performing HARP analysis improves the performance of cardiac motion estimates from tMRI.

## Conclusion

The simple prior time-reversal operation applied to a systolic image with the forward model of the LV wall motion accounts for the large displacements experienced by the myocardial tissue in sequentially-acquired tMRI data. The remaining smaller magnitude regional myocardial motions in the time-reversed image can be computed by an estimation technique selected by the user. Using this two-step approach, we showed that HARP analysis became more immune to phase wrapping and consequently the motion estimation performance is enhanced. Robust measurements obtained with our approach therefore allow better interpretation of the cardiac motion. This should enable computation of accurate strain fields to characterize the regional deformation aimed at evaluating the function, viability and pathological state of the underlying myocardial tissue.

## Authors' contributions

TA helped with data acquisition and performed numerical simulations and computations. IVS participated in the design and coordination of the study. LTC participated in modeling the LV wall motion. MB conceived of the study, participated in its design and coordination, interpreted the results and drafted the manuscript. All authors read and approved the final manuscript.

## Appendix A. Preprocessing of tMRI data

**Figure 12:**
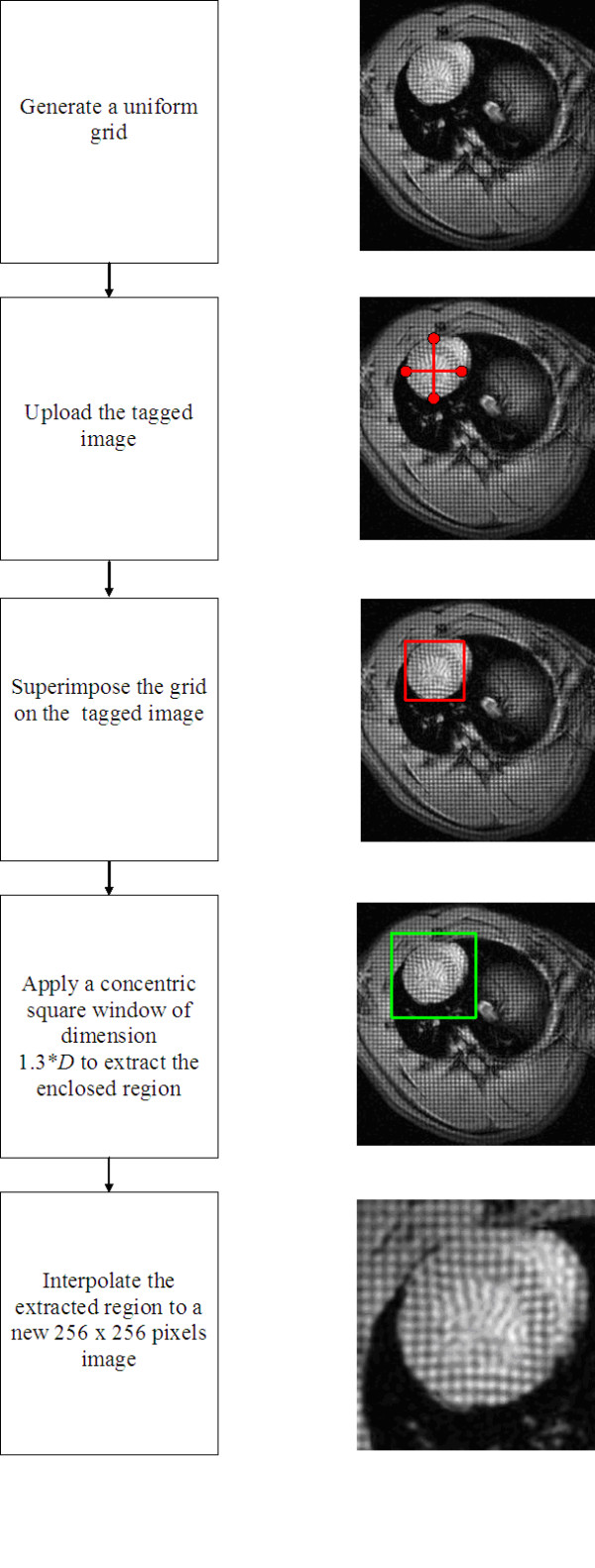
The procedure used for segmentation of the left ventricle from a larger tagged image is described algorithmically.

A set of image processing routines was implemented for application on the tMRI data prior to performing the motion analysis. The main task of these routines was to ensure that the resulting model was generic enough to either represent the myocardial motion in different species or account for the variations seen in the heart volumes within the same species under normal or pathological conditions. Application of this procedure aimed to segment out a portion of the acquired tagged image to visualize the LV in a larger short-axis view at the mid-ventricle. This segmentation process is given in detail in Fig. 12. As shown algorithmically in the figure, the end-diastole image, which was acquired when the LV is fully open as the first image of the systolic phase, was displayed on the computer screen. Next, four points that were 90 degrees apart were selected on the outer circumference of the LV wall. Then, two crossing lines, each passing through two of the selected points, were drawn. The cross point of the two lines was set to represent the center *C *of the LV. The average of the lengths of the two lines was used as a measure of the LV diameter *D*. Next, a square window with equal size *D *in each dimension and centered at *C *was drawn to distinctly enclose the LV wall. A second concentric square window was applied with a slightly larger dimension *N *= 1.3*D to extract a broader region containing the LV wall of the heart and the surrounding tissue mass. This last window was also used to segment out the LV from the remaining systolic image frames *F*_*i *_for *i *= 2,3 ... 5. Finally, the extracted images from all frames were interpolated to an increased dimension of 256 × 256 pixels to obtain the final systolic image set *I*_*i *_for *i *= 1,2 ... 5. With this standardization outlined and depicted in Fig. 12 as a flowchart, the displacement measurements are now expressed in terms of the pixel units. Figure 1 shows a set of images obtained after the application of this segmentation routine followed by a spatial scaling of the image coordinates.

## Appendix B. A simple model of the LV wall motion in short-axis view of heart

Current research efforts are focused on spatio-temporal modeling of the LV shape and motion. To aid these efforts and provide a model for the time-reversal operation in the main body of the paper, here, we introduce a mathematical construct as a means of quantitatively describing the LV wall motion in a beating heart.

### Anatomical and structural characterization of the LV

For clinical analysis or evaluation purposes, the short axis view of the heart is divided into three sections, namely base, mid-ventricle and apex. This division is in accordance with the standardized myocardial segmentation published by the American Heart Association [21]. To perform its pumping task, the LV and its myocardial fibers are organized in a complex architecture both anatomically and structurally within these three levels [17]. As a result, the LV wall twists with respect to its long axis at the base and apex in opposing directions, and consequently the deformations at these levels are governed by torsion together with a combination of radial and circumferential strains. The motion at the mid-ventricular level, on the other hand, is relatively twist-free and hence the deformation in this region is described mainly by the radial and circumferential strains. Moreover, the patterns and degrees of the LV wall motion are also known to depend on the size of the ventricle at the level observed. At the level of the mid-ventricle, the LV cavity diameter experiences its greatest radial shortening (elongation) during the systolic (diastolic) phase. If all aspects of the LV motion are considered, prior knowledge and experimental observations suggest that its complete characterization with a model would be a challenging task to undertake. Nevertheless, if the mid-ventricle is chosen as the specific level of interest, a simple yet powerful model can be constructed. Due to the minimal torsion effects at the mid-ventricle, we propose a model that is capable of describing the LV wall motion comprehensively at this almost twist-free level.

Before proceeding further, we make the following basic assumptions for constructing the model to properly represent the LV motion at the mid-ventricle.

1) The myocardial tissue deforms the same manner whether it is in the septum or outer LV wall.

2) Although a full cardiac cycle includes both systolic and diastolic phases, to keep the analysis simple, the model is built to mimic the systolic LV motion only. Nonetheless, it is straightforward to extrapolate the analysis to include the diastolic phase if desired. The digital cardiac images acquired sequentially in equally spaced time intervals in the systolic phases are identified by the frames *F*_*i *_for *i *= 1,2 ... 5, where *i *= 1 is the end diastole frame and *i *= 5 is the end systole frame.

3) The cardiac images are constructed on a virtual canvas with rubber sheet properties that can be digitally stretched according to a spatially varying deformation pattern. With this elastic feature, the canvas serves as a basis upon which the model acts through the application of a spatial perturbation in the coordinates of the myocardial tissue. Thus, such a spatial transformation describes the local myocardial motion. As shown below, the canvas' elastic behavior is exploited through the use of time-forward operations applied on the images to simulate the real myocardial motion.

### Motion model of the LV

The mathematical basis for modeling the LV motion in the current work has been described in [22]. The model involves Gaussian function for deforming the image canvas to simulate the forward systolic motion of the LV wall during the cardiac cycle. The resulting effect by such deformation is equivalent to shifting the image coordinates under a spatially variant coordinate transformation, thereby making the transformed state resemble the myocardial contraction observed during the systole. Mathematically, the motion model can explicitly be expressed by the coordinate transformation **T **defined by

(B1) $$T=\left(\begin{array}{cc} 1+e^{\frac{-(x^{2}+y^{2}}{{\mathrm{)\alpha}}^{2}}} & 0\\ 0 & 1+e^{\frac{-(x^{2}+y^{2})}{\alpha^{2}}} \end{array}\right).$$

**Figure 13:**
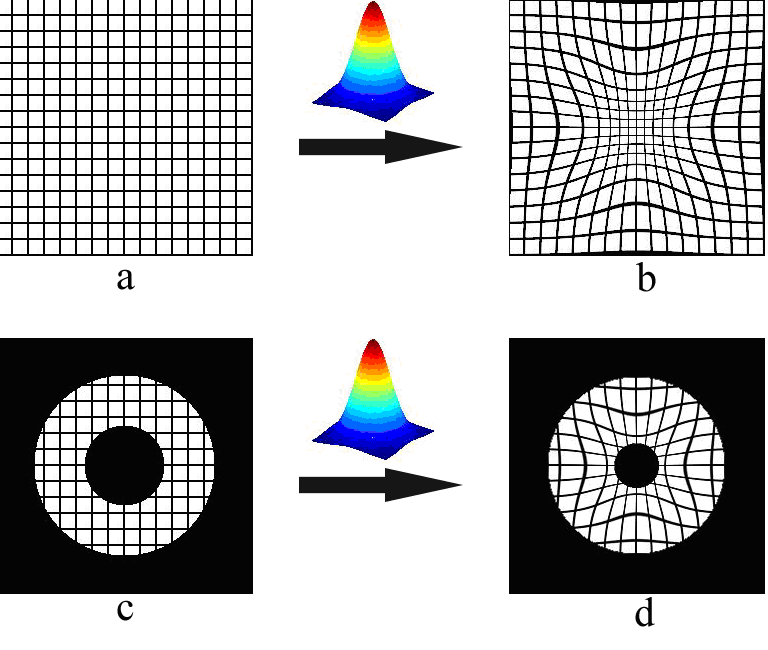
**A uniform image *I*(*x, y*) in figure a is labeled with regular grid tags.** The operation **T **[*I*(*x, y*)] yields the deformed image in figure b, where the shape and size of the tag cells depict regional variations in motion. Similarly, a donut-shaped disk in figure c is used as a simple model of the left ventricle. Figure d shows the resulting deformed disk.

Here *x *and *y *represent spatial coordinates. The parameter *α *in the exponent is responsible for the amount of radial deformation applied on the canvas, and is therefore time dependent when the serially-acquired image frames are considered. The real cardiac tMRI data are used to estimate *α*_*i *_in characterizing the LV motion from the first frame *i *= 1 (*α *→ 0) to a selected frame *I*_*i*_, *i *= 2,3,4 or 5, in Fig. 1. Figure 13 illustrates the application of this transformation to canvases of two tagged images. A uniform image *I*_1_(*x, y*) in Fig. 13-a is labeled with regular grid tags. The operation **T **[*I*_1_(*x, y*)] yields the deformed image *I*_2_(*x, y*) in Fig. 13-b, where the changes in shape and size of the tag cells depict regional motion. If a donut-shaped disk in Fig. 13-c is used to mimic the LV wall, Fig. 13-d shows the resulting deformed disk. The radial LV wall motion is directly related to the value of *α*; small *α *yields small wall motion and large *α *produces large wall motion.

To accurately describe the myocardial motion, it is necessary to satisfy two main criteria observed in the MR experimental data, as discussed in the literature. First, the transformation function should exhibit radial dependence, so that image coordinates closer to the LV cavity's center are deformed more than those further away from the origin. This criterion is clearly satisfied in Figs. 13-b and 13-d. The bow-shaped behavior exhibited by the deformed lines indicates that the resulting deformation has radial dependence. Second, the transformation function causes an increase in the thickness of the LV walls, and a decrease in the size of the LV cavity as observed in the real systolic cardiac motion. This requirement is also met by the proposed model. The resulting donut in Fig. 13-d clearly exhibits increase in the wall thickness, and decrease in the cavity size, which are both desired features to accurately model the LV motion. In these regards, the parameter *α *alone can be seen as capable of mimicking the changes in the LV wall's diameter and thickness.
